# What does current science tell us about the accuracy, reliability, and completeness of intoxicated witnesses? A case example of the murder of a prime minister

**DOI:** 10.3389/fpsyg.2022.982992

**Published:** 2022-10-28

**Authors:** Malin Hildebrand Karlén, Andrea de Bejczy, Henrik Anckarsäter, Gísli Guðjónsson

**Affiliations:** ^1^Department of Psychology, University of Gothenburg, Gothenburg, Sweden; ^2^Department of Psychiatry and Neurochemistry, Centre for Ethics, Law and Mental Health, Institute of Neuroscience and Physiology, The Sahlgrenska Academy, University of Gothenburg, Gothenburg, Sweden; ^3^Department for Forensic Psychiatry, National Board of Forensic Medicine, Gothenburg, Sweden, Gothenburg, Sweden; ^4^Department of Psychiatry and Neurochemistry, Institute of Neuroscience and Physiology, The Sahlgrenska Academy, University of Gothenburg, Gothenburg, Sweden; ^5^Institute of Psychiatry, King's College London, London, United Kingdom; ^6^School of Business, Reykjavík University, Reykjavík, Iceland

**Keywords:** blood alcohol concentration (BAC), witness psychology, intoxicated witnesses, alcohol intoxication, alcohol consumption levels, modified Widmark equation, cognitive impairment alcohol

## Abstract

Generally, the testimony of intoxicated witnesses has been considered relatively unreliable, but recent research has nuanced the knowledge base regarding these vulnerable witnesses.

**Purpose:**

To demonstrate the application of recent research findings regarding intoxicated witnesses to the statements made by a key witness to the murder of Olof Palme, Sweden's prime minister, in 1986. An additional purpose was to illustrate the use of a nuanced calculation of blood alcohol concentration (BAC) for researchers.

**Methods:**

The Palme murder has been debated since the crime was committed and no one has yet been sentenced. One of the witnesses was intoxicated and to estimate a range for his BAC at the time, a comprehensive BAC calculation was conducted in this study to illustrate important factors to consider in these types of cases.

**Results:**

Through the demonstration of the use of a nuanced BAC formula and by applying recent research results from studies on intoxicated witnesses, it was estimated that the possible BAC of the witness in the Palme-case at the time of the witnessed crime ranged between BAC = 0 to BAC = 0.13, depending on the type of alcoholic beverage consumed and whether the witness was a social or heavy drinker. This puts the witness either well within the span of maintained completeness as well as maintained accuracy rate (if considering: lowest dose and heavy drinker), or slightly exceeding this span into the BAC-range of reduced completeness but maintained accuracy rate (if considering: highest dose and social drinker). He was questioned immediately, and thereafter repeatedly, and he reported similar information throughout the interviews, which is in line with previous results on information maintenance over repeated interviews among intoxicated witnesses.

**Conclusion:**

The current case example shows how recent research on intoxicated witnesses can be applied in praxis, illustrating important factors for legal practitioners to consider when interpreting information from intoxicated witnesses. It also provides legal practitioners and researchers with an example of a structured approach to more nuanced BAC-calculations.

## Introduction

On 28 February 1986 at approx. 23:00 PM, the Swedish prime minister Olof Palme was murdered in the open street in central Stockholm. Now, over 35 years later, motives for the murder and the perpetrator's identity are still debated. Many witnesses have been questioned, but only a few are key witnesses in the sense that they witnessed the murder from a close distance. One of those witnesses, the case example in focus in this article, was standing close to Palme and his wife at the time of the murder, but the reliability of his testimony could potentially have been undermined by his preceding alcohol consumption. The first aim of the present study was to, using the Palme case as an example, illustrate how recent research on alcohol intoxicated witnesses could be applied to assess the reliability of information within the decision-making processes in criminal investigations where intoxicated witnesses play a central role. The second aim was to demonstrate the use of a more nuanced BAC-calculation than is often used in applied studies on intoxicated witnesses, outlining the principal factors to consider that influence the BAC-level.

### Alcohol's impact on perception and memory in a witness context

The degree of alcohol's impairment of cognitive functions is dose dependent, causing successively increasing negative effects on many cognitive functions, such as focused attention, divided attention, and working memory capacity (Sayette, 1993; Curran, 2006; Zoethout et al., 2011; Dry et al., 2012). In a systematic review of 19 empirical studies that had examined the impact of alcohol on memory in forensic contexts (Flowe et al., 2021), three crucial areas of memory performance were identified: *memory accuracy* (i.e., the ability of the witness to distinguish between correct and incorrect information pertaining to the crime); *memory reliability* (i.e., the credibility of the witness in terms of how safe it is to rely on their evidence); and *completeness* (i.e., the quantity of information provided). In line with a meta-analysis (Jores et al., 2019), the review by Flowe et al. (2021) showed that acute alcohol intoxication (mean BAC-levels in most studies = 0.03–0.09) deleteriously affected the completeness of the information, but not its accuracy or the reliability of what had been remembered.

This is hardly surprising since, despite such findings, the reliability of intoxicated witnesses' ability to perceive and recall events has long been questioned within the judicial system (Benton et al., 2006; Evans et al., 2009). Alcohol has a broad and complex impact on cognitive functioning, affected by factors such as time elapsed between the last drink and witnessing the event, drinking pace, alcohol concentration, and the person's alcohol tolerance (i.e., high/low consumer). Alcohol has both stimulating and sedating effects, and cognitive impairment can be related to both (Hendler et al., 2013). Typically, when the BAC curve is rising, alcohol's effects are stimulating, whereas on the descending slope alcohol typically has a sedating effect. There are individual differences in the stimulant and sedative effects of alcohol, but generally, when BAC >0.08g/kg (e.g., BAC = 0.08–0.15), alcohol's effects are sedating, diminishing the capacity for maintained attention and self-reflection, while lower doses are generally stimulating and make a person more talkative, confident and less inhibited (Söderpalm, 2011 and WHO, 2022). Also, a more extensive memory impairment of alcohol intoxication on ascending limb of BAC-curve has been noted, where not only recall is impaired but also recognition (see Söderlund et al., 2005). Tolerance has an impact on these effects, for example, individuals with a high risk for alcohol dependence seem to experience a reduced sedative component (Hendler et al., 2013).

### Alcohol intoxication and memory

Based on the negative impact of alcohol on several cognitive functions crucial for encoding information (e.g., maintaining attention, organizing information), it should not be surprising that consuming alcohol before encoding an event often disrupts later recall, both in basic laboratory studies on memorizing lists of words and in applied forensic studies using interviews (Mintzer, 2007; Zoethout et al., 2011; Jores et al., 2019; Flowe et al., 2021). However, research overviews (e.g., White, 2003; Mintzer, 2007) have emphasized that BAC:s <0.15 generate only small to moderate impairing effects on memory. In previous basic memory research, low alcohol doses (i.e., ≤0.3g/kg, approx. ≤0.03) have had no effect, very high doses (i.e., ≥ 2.0g/kg, approx. ≥ 0.2) a clear impairing effect (Goodwin et al., 1970; Hashtroudi et al., 1984; Zoethout et al., 2011). In previous studies, alcohol levels were ~≥0.8‰ have more consistently resulted in memory consolidation problems, for example in the forms of greyout/blackout. During the course of an alcohol-induced blackout/greyout, information can be retained and recounted as long as the information is still actively processed in working memory (Curran, 2006), but consolidation into long-term memory storage is impaired (i.e., greyout: information can sometimes be recalled after specific prompting) or non-existent (i.e., blackout: information cannot be recalled at all) (see Birnbaum et al., 1978; Curran, 2006). A dose of approximately 0.8g/kg (~0.8‰) has been shown sufficient to decrease episodic recall performance, in basic memory studies (Birnbaum et al., 1978: 0.7g/100 ml, BAC = 0.08; Tracy and Bates, 1999: 0.8ml/kg, BAC = 0.08) and in applied studies on alcohol intoxicated witnesses (e.g., Schreiber Compo et al., 2012; Flowe et al., 2016; Karlen et al., 2017). Within these cited studies, as in the review by Flowe et al. (2021), it is important to note that the accuracy rate did not decrease at these BAC-levels.

### Interview timing and repetition: Completeness, accuracy, and reliability

Another important aspect to consider in the intoxicated witness' situation is factors pertaining to interview conditions. First, not all witnesses are interviewed directly after the event (Evans et al., 2009). The intoxicated witness in the Palme case gave an initial statement at the scene but was not formally interviewed until later that same night. Thereafter, he was interviewed repeatedly during the course of ~1 year (in total, he gave three interviews and one initial statement, see Appendix). Therefore, his statements, as in the majority of similar cases where serious crimes have been committed, could be affected by both the effects of (a) delay before the interview and of (b) repeated questioning, on memory processes and recall ability.

#### Delayed interviews

It is well known that memory decays with time. Research indicates (Yuille and Tollestrup, 1990; Hagsand et al., 2012; La Rooy et al., 2013; Karlen et al., 2017), in line with the assumptions of many police officers (Evans et al., 2009) that witnesses, despite a certain degree of intoxication, tend to report the most, and slightly more reliable, information soon after the event. Actually, in previous applied studies on intoxicated witnesses (e.g., Karlen et al., 2017) the effect of time generated the same pattern of decreased completeness and maintained accuracy rate, but in fact with an even stronger effect, than intoxication (BAC ≥0.08) did. Furthermore, indications in previous research are that alcohol-induced cognitive deficiencies during encoding could be magnified by time-induced memory decay. In other words, if a witness is intoxicated rather than sober, the risk is greater of even shorter reports 1 week later than what had been the case for a sober witness (i.e., due to time-induced memory decay alone) (see Karlen et al., 2017). Therefore, for maximizing the amount of salient information provided, it may be of particular importance to interview intoxicated witnesses immediately after an observed event.

#### Repeated interview

Even though memory decays with time, repetition seems to some extent counteract this effect (Baddeley et al., 2011) even for intoxicated witnesses. The beneficial effect of repetition is contingent on the amount and quality of rehearsal (Rundus, 1971; Woodward et al., 1973; Craik, 2002), which alcohol can impair (White et al., 2000). However, previous applied studies where witnesses were moderately intoxicated both during the event and the immediate interview, show a beneficial effect of repetition in counteracting memory decay over subsequent interviews (i.e., repeated interviews) (Yuille and Tollestrup, 1990; La Rooy et al., 2013; Hagsand et al., 2017). Despite intoxication, these witnesses reported more information, as well as more reliable information, 1 week after the event if they also had given a direct interview. This lends support to the assumption that enhanced focus/repetition of the event shortly after the crime facilitates long-term memory encoding, despite the levels of intoxication used in previous studies.

### Quantity-accuracy trade-off

As a general theoretical framework, the quantity-accuracy trade-off offers a plausible explanation for both the above-reported relationship between the amount *vs*. accuracy of the information, as well as for the long-term beneficial effect of repetition. Accuracy has often been preferred before quantity in studies of interviews performed by witnesses under high cognitive load (Pansky and Nemets, 2012). Since accuracy did not decrease in these studies, this lends support to a general preference for accuracy over quantity when intoxication rises high enough to force a ‘choice' between the two (i.e., BAC ≥ 0.08; for a discussion, see Karlen et al., 2017). Results from a meta-analysis on intoxicated witnesses (Jores et al., 2019) and review (Flowe et al., 2021) also support this conclusion; lower degrees of intoxication do not seem to require making such a ‘choice' (i.e., no differences have been found in quantity-level between the low degree of intoxication and sober states), but higher BAC-levels do. The proposed function is that with rising BAC-level, intoxication produces successively increasing cognitive load. At a basic level, this can be related to successively decreasing neuronal firing, particularly in the hippocampus, as a consequence of rising BAC-level (e.g., White and Best, 2002), which would strain cognitive processing and memory encoding ability. However, at a certain point, this BAC-related impairment would be strained to a point of making encoding of new memories not only difficult or partial, but impossible, and the person subsequently unable to recall anything during this critical period. This decreases the scope of the witnesses' cognitive abilities to perceive, associate, and encode incoming information.

Based on previous studies on alcohol intoxicated witnesses' free recall, motivated, non-biased alcohol intoxicated witnesses (at the researched levels of intoxication) seem to be able to choose to report details they are certain of and leave out less reliable details. Hence, this is in line with research on what pattern should emerge when the interviewee strives to report as correctly as possible during high cognitive load (Koriat and Goldsmith, 1996; Koriat et al., 2000). The quantity-accuracy trade-off is also relevant to other aspects of the situation that witnesses to violence often are in, as in the Palme-case, where stress and divided attention (i.e., due to increased cognitive load) make it harder to process incoming information in an analytical and situation-oriented manner. This can lead to favoring short-term outcomes and neglecting long-term consequences (Josephs and Steele, 1990; Deffenbacher et al., 2004).

#### Conclusions from applied studies on intoxicated witnesses

The research field of intoxicated witnesses is relatively young and the applied studies to date have shown mixed results regarding their completeness and accuracy (i.e., overall reliability). This can, at least in part, be attributed to the use of different designs and alcohol doses, as well as to varying interview formats and timing (see also Jores et al., 2019 for a similar conclusion). However, decreased completeness and maintained accuracy has been rather consistent in studies that have used (a) an immediate interview, (b) alcohol doses generating BAC:s ~0.06–0.10/~0.6–0.8‰, and (c) a relatively open interview format (i.e., free recall or open questions) (e.g., Flowe et al., 2016, 2021; Karlen et al., 2017; Jores et al., 2019). Even though delaying the interview seems to reduce report completeness for both intoxicated and sober witnesses, intoxicated witnesses seem to be more negatively affected by this (i.e., report even less information in delayed interviews). Nevertheless, such a delay did not seem to affect accuracy in these studies to a considerable extent (80 vs. 85% in Karlen et al., 2017), leading to conclusions that under free recall circumstances, a statement given one week after the event can be as reliable (alternatively with a minor decrease in the proportion of accurate details) as a statement given directly after the event (Yuille and Tollestrup, 1990; Hagsand et al., 2017; Karlen et al., 2017).

### The present study

The overall purpose of the present study was to contribute to bridging the gap between research and legal praxis regarding alcohol intoxicated witnesses. The first aim was, by using the intoxicated witness in the Palme case as an example, to illustrate a practical application of recent research on intoxicated witnesses to violence—how could these research results inform the decision-making of legal practitioners within criminal investigations? Based on the application of research results to this case, a summary of some important factors was made concerning interviews with intoxicated witnesses and the evaluation of their statements. The second aim was to, again by using the circumstances of the witness in the Palme case as an example, illustrate how to conduct a more structured analysis of BAC impact on witness statements than what has generally been done in recent research on intoxicated witnesses. This was done both by utilizing a more nuanced equation and by highlighting additional factors that are especially relevant to consider when assessing BAC-level.

## Methods

The present study was based on a detailed review of interview material from the Palme-case, given by a key witness who was alcohol intoxicated at the time of the murder. A detailed analysis of the statements and the application of the BAC calculation on the estimation of completeness, accuracy, and reliability was conducted, relating this information to the circumstances during which he witnessed the crime. The results were discussed in light of recent developments within the research field of intoxicated witnesses. Also, to illustrate how to calculate BAC for intoxicated witnesses in a nuanced manner, a step-by-step analysis of his BAC was performed. In the analysis, using a modified Widmark equation, all available parameters relating to alcohol intake, individual variables, and relevant circumstances in the witnessed situation were considered.

### Material and procedure

The witness gave in total of three interviews (documented) and one initial statement (not documented), which took place between 1986-02-28 and 1987-03-25 (with an addition made 1987-08-07 to the last documented interview after telephone contact). All interview transcripts from interviews conducted with this witness were obtained in their entirety from the archives of the Swedish Police Authority. The statement the witness gave directly at the scene of the crime was not included in the documentation of the Palme-case within the Swedish Police Authority's archives, and it is, therefore, unclear whether it had been documented at all by the police. That the witness had made such a statement was mentioned in the transcript of his subsequent interview, which was conducted later that same night. All transcripts were written as a summary description (i.e., not documented verbatim) and varied in degree of detail, regarding what the witness had reported. An overview of all documented interviews and interview units included in the analysis are presented in Appendix.

The documented interviews were analyzed using a qualitative approach, a content analysis, which is a type of thematic analytical approach. Specifically, as opposed to more traditional forms of thematic analysis, the purpose of content analysis is to summarize and structure information in overarching categories without interpretation. This approach is often used in medical research where information is gathered from medical journals (see Graneheim and Lundman, 2004). After reading through all documented interviews, information units were selected for analysis from the documented interviews (for transparency, all selected interview units for this analysis were presented in Appendix). This selection was guided by results from previous research on intoxicated witnesses, each information unit in this documentation was considered whether it was deemed relevant to the analysis in the present study (i.e., factors that according to previous research on alcohol intoxicated witnesses could have influenced the completeness, accuracy, and reliability of his report). Particular focus was on information describing his degree of intoxication, contextual factors the witness presented, information regarding interview timing and the conducting of repeated interviews, as well as the interaction between these three factors. Hence, the focus within the content analysis of this interview material was on identifying circumstances particularly relevant when evaluating information from an alcohol-intoxicated witness to violence, given in interviews: (a) while still in an intoxicated state (i.e., later the same night), and (b) in a sober state, issues associated both with delay and repeated questioning (i.e., ~2 days later, as well as 13 months thereafter). Based on these documented interviews with this intoxicated witness to the Palme murder, important factors regarding his cognitive state due to alcohol (and potential moderating factors such as stress, fatigue, and darkness) were identified and these factors were evaluated in light of recent research on how intoxication may alter witnesses' perception and memory. A description of how such factors may have affected the witness's ability to perceive information, encode it in long-term memory, and recall (i.e., retrieve) the information in interviews, was given, as well as discussing potential pitfalls within the relationship between suggestibility, alcohol intoxication and stressful circumstances relevant to the case.

### The Palme-case: Relevant case background factors and witness research

Olof Palme was shot to death at 23.40 PM the 28 February 1986. General contextual factors of importance for this content analysis were chosen based on previous research on eyewitnesses and alcohol's effect on perception regarding the crime. These contextual factors were that apart from Palme being a famous person in Sweden and this consequently a high-stakes case for all involved, it was dark, did not rain, and the murder was committed on a street in central Stockholm (i.e., a relatively well-lit scene despite being a Swedish winter night). It was cold, with many people wearing coats and hats, and although not extremely busy, this was a Friday night (i.e., most likely more people being out in bars and restaurants at this time of the evening than on another weekday). The witness walked 3–7 m (this fact varies somewhat between interviews) behind the Palme couple and the murderer for a short while (approximated by the witness as seconds rather than minutes). Arguments for general poor witness ability in the present context are, (a) the darkness, (b) the short time frame between the witness starting to walk behind the couple and the murder being committed (c) the likelihood that the witness was not fully focusing his attention on the Palme couple since he said that he did not look at them the entire time and did not recognize the persons walking in front of him. Two ameliorating factors in this situation, again based on previous research on general factors, are, first that streetlights were present, and second, the witness walked close behind the couple and the murderer.

## Results

### Key points in his statements: Consistencies and variations over interviews

#### Times of interview and repetition

The initial statement was given by the witness as soon as the police arrived at the scene (i.e., shortly after he witnessed the murder). According to information documented in his first interview, later that same night, he said to have “told what he knew” to a female police officer at the crime scene. After having given this initial statement, he had gone home. He said in a later interview (03-03-1986) that he had talked to his wife when he arrived home and had said to her that the man had a dark blue roughly knitted cap on which covered his ears and had a wide upwards fold by the cap's edges. He also said to her that he was certain about the facts regarding the cap. That he had indeed said these things was confirmed by his wife at the time of this interview. The wife also added that she had perceived her husband at the time to be “incoherent in his account of the event” which she presumed was due to the that he seemed to have had a shock and partly that he seemed to be alcohol intoxicated. The factual information he recounted to the police during the first documented interview was the same as that he told his wife when he came home shortly after (i.e., on the night of the murder) (see Appendix for full details). Considering this sequence of events in light of recent research on intoxicated witnesses regarding the aspect of repetition, this witness had gone through some procedures previously shown beneficial for memory consolidation. He had given an immediate statement as well as recalled/repeated these events later that same night when talking to his wife. The first proper interview at the police station (i.e., the first interview after his immediate statement) was conducted later that same night. This means he had not only the opportunity to rehearse his recollections of the events by himself but also through reporting them to other persons at least two times (incl. the police officer and his wife) before being interviewed properly by the police for the first time later on the same night of the crime. The second proper interview with this witness was conducted during the evening the following day (i.e., in a sober state, ~20 h after the crime). The second interview was conducted on a Monday, during which it cannot be ruled out that he had talked about this event again with somebody or several persons, especially considering the great national interest in this crime.

#### Indications of uncertainty

He displayed some evidence of partial amnesia (i.e., periods of time which he did not recall) as well as memory confusion (i.e., memory fragments that were recalled but could not be placed in space: e.g., who wore the dark coat; or time: e.g., how long time had elapsed). Furthermore, his statements regarding certain details of an observed coat varied somewhat between the first (i.e., the same night, still under the influence of alcohol) and second interview (i.e., 3 days later, sober state), with the coat being said to go below the knee the first time and “to the knee” the second time. However, no direct alterations of previously reported information were found.

#### Indications of reliability

The witness was generally consistent in reporting information, for example, that he according to his wife, gave the same factual information to her when coming home that night, during the first interview, and during the second interview (i.e., this was the first interview given in a sober state). Also, despite expressing uncertainty regarding temporal aspects, his general approximation of time did not seem significantly impaired since he thought his company left the restaurant around 11.00 PM and CCTV records confirm that they were at the ATM a quarter of an hour later (i.e., self-reported information regarding time is corroborated by objective facts). Between interviews two and three, he was consistent regarding the number of persons in his company who left the restaurant in question to go to another pub/restaurant, which people in the company who fell behind and why, and the names of the persons in the company who walked with him. Taken together, despite partial amnesia and some source memory confusion, certain information seemed to be reliably reported over interviews, (i.e., 2 days after vs. over 1 year after the crime), and his temporal approximation was also relatively intact. However, minor inconsistencies emerged between the second and third interviews. In the first and second interviews, he said all three persons were of the same height, while in the third interview, he describes the shooter and Palme to be of the same height while the woman was shorter. In the first and second, he talked about walking behind the three persons for a few meters, and also that the shooter had his arm around Palme, while in the third interview, he claims to be unsure of if he had walked behind them or if they suddenly walked out in front of him as well as that the shots had been fired as soon as the shooter put his arm around Palme.

### Alcohol profile: Example of BAC-estimation for this witness

**Figure 1:**
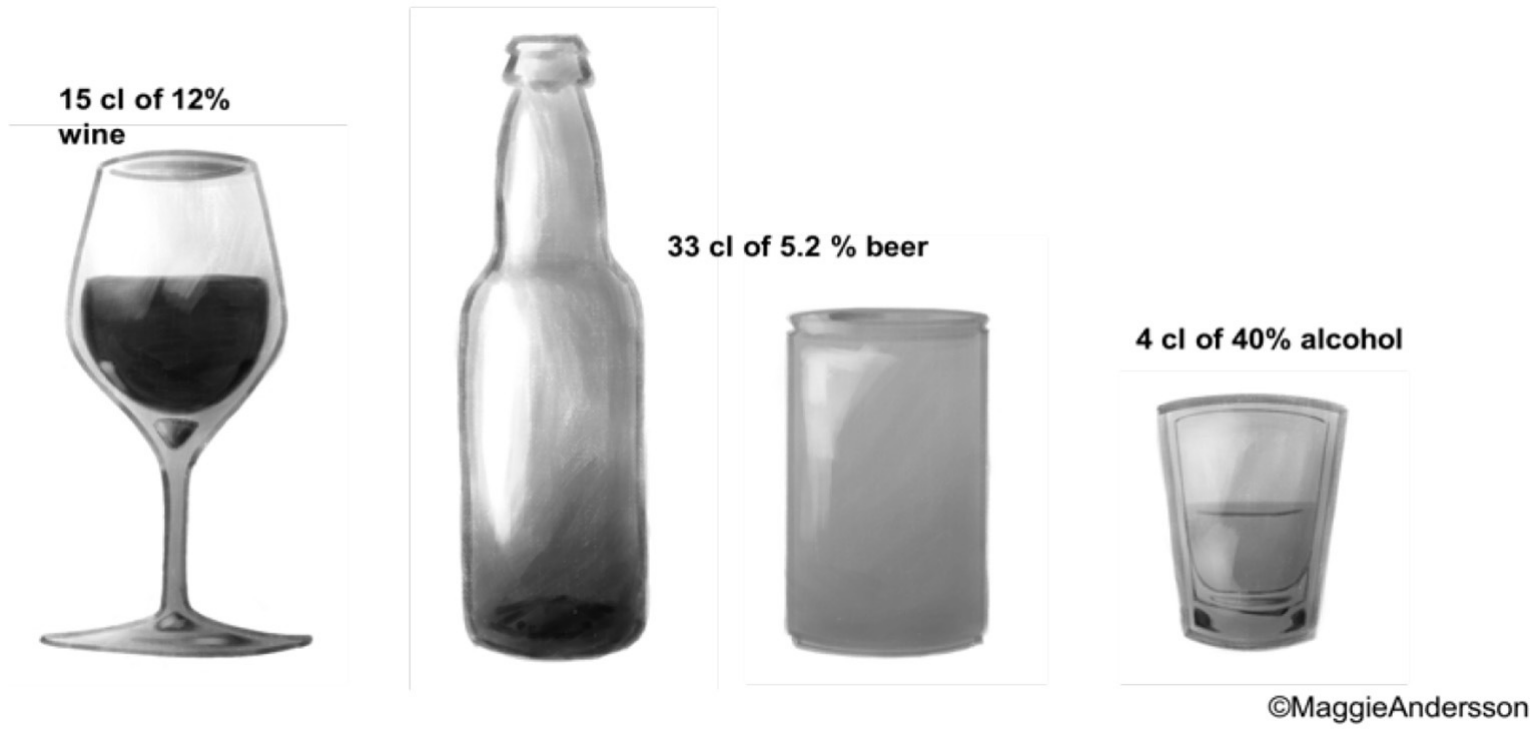
Examples of one unit of alcohol (12–14 g). One can/bottle of 5.2% beer; 33cl/12fl oz =13.5 g alcohol, one glass of 12% wine; 15cl/5fl oz =14 g alcohol, one shot of 40% hard liquor; 4 cl/1.5 fl oz% =12.6 g alcohol. Reproduced with permission from Maggie Andersson, private commission.

The definition of one unit of alcohol differs substantially between countries, ranging from 8 g of alcohol in UK, to 20 g in Austria. The most commonly used definition in research is 14 grams, as in the US definition (Kalinowski and Humphreys, 2016) (see Figure 1 for examples of a standard drink).

To make the estimation of the present witness' BAC-level as accurate as possible, several factors need to be considered. Based on the information from interviews, important factors regarding BAC calculations and interpretation of estimated BAC are age (here: 39 years), height, and weight (of average height and length or possible somewhat shorter and heavier based on descriptions of him and on a photo from a re-enactment during the police investigation); level of consumption (described as a social drinker); physical activity (no physical exercise effect apart from the ~25-min walk from the restaurant to the scene of crime); use of any other drugs or medications (none documented at the time); food intake (consumed dinner during the evening); type of beverage (mixed). However, the most important factors are the amount of alcohol consumed, percentage of alcohol consumed, and time frame of consumption. The witness reported in Interview 1 that he drank alcohol during an after-work dinner. When asked, he couldn't recall exactly when he started or finished drinking. He reported in Interview 2 that he and his friends from work had been at a restaurant and stayed there between ~5.00 and 11.00 PM, and told the police how much he had been drinking. In the documentation of Interview 3, it is noted that the witness reports the time between 4.30 and 11.00, and on the following date 07-08-1987, had specified this information, reporting that he had been drinking three shots and three beers during “happy hour” (here: between 4.30 and 6.00 PM) and that he thereafter possibly had had one whisky and/or “sipped on one beer during the rest of the evening”.

**Figure 2:**
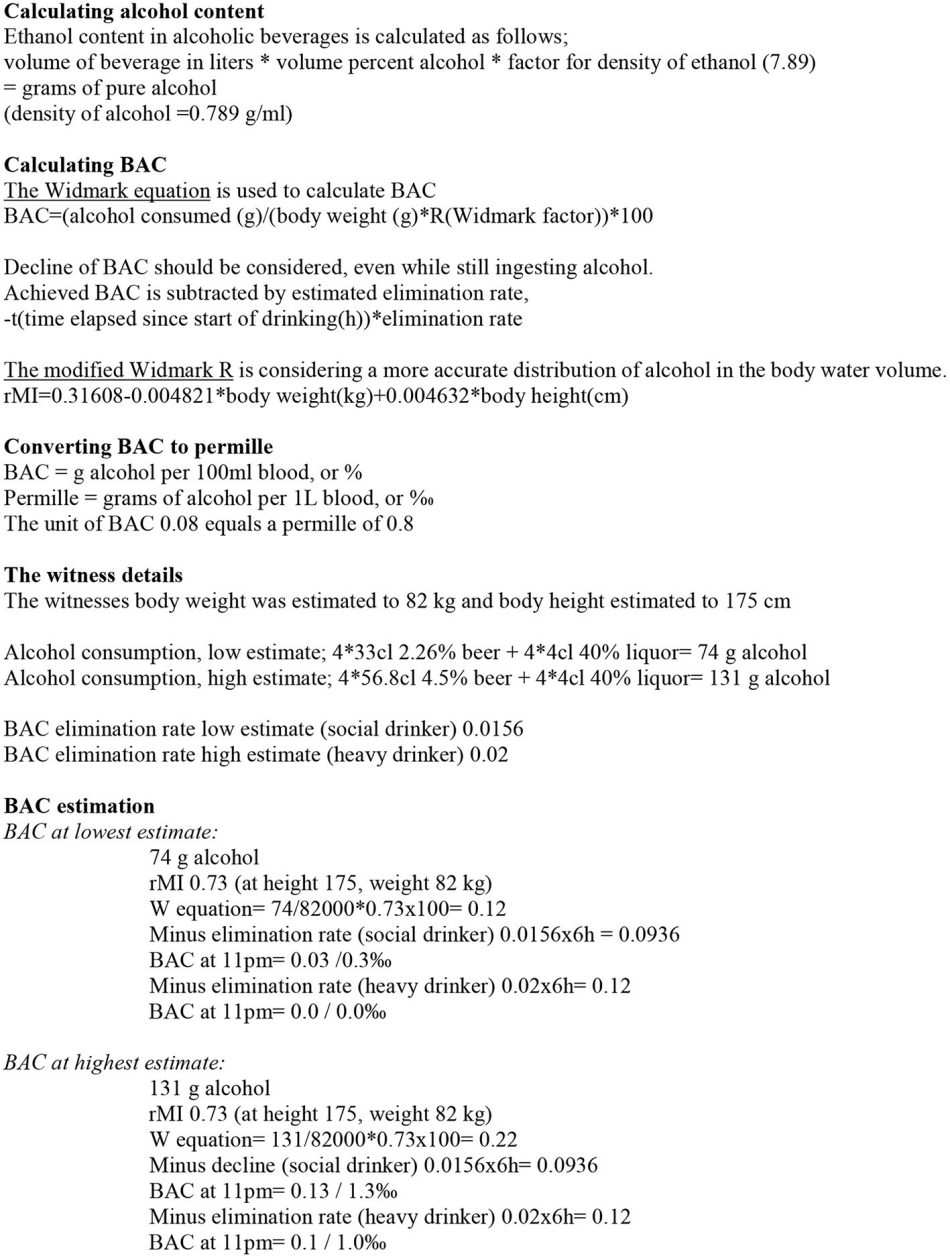
Calculation of possible BAC-range.

For the calculations of the present example analysis of BAC, we assumed the numbers of drinks during the evening to be: 3^*^4 cl 40% spirits and 3 beers (in the range of 33–56.8 cl and 2.26–4.5‰), which were consumed during ~1–1.5 h. Plus the one beer (in the range of 33–56.8 cl and 2.26–4.5%) and the additional whisky (4 cl, 40%) possibly consumed after the substantial dinner at 8 PM. The time frame for alcohol consumption used in the calculation is 6 h, starting at 5 PM and witnessed crime at 11 PM. Regarding the kind of alcohol consumed, a normal alcohol absorption rate, or possibly on the slow side, was assumed (Paton, 2005). The influences of food on BAC absorption and elimination are hard to estimate. In this case, the dinner consumed ~2 hours after the three shots and three beers were consumed during ‘happy hour' could have had an effect on BAC (Finnigan et al., 1998; Norberg et al., 2003). However, since only “dinner” is mentioned as a description of his food intake without details regarding amount or content (and since it is not possible to include food as a factor in the BAC calculation), the effect of food intake on alcohol absorption will be limited to the interpretation of the impact of the estimated BAC. As atypical variants of alcohol metabolizing enzymes are uncommon in Caucasians, the alcohol consumed is assumed to be metabolized at the rate most common among Caucasians (Wall et al., 2016) (see Figure 2 for an overview of the calculation of BAC range).

For the purpose of the BAC calculations, the start of alcohol consumption was set to 5.00 PM (based on his report of “happy hour”). The majority of alcohol was consumed for the duration of 2 h and peak BAC was probably reached ~6.30–7.00 PM. The average height and length for men in 1986 were 179.8 cm and 78.2 kg, resulting in BMI = 24.2 (Statistical Central Bureau, Sweden). Assuming that the witness was slightly shorter and of a slightly heavier build (according to description), the estimates of 175 cm and 82 kg were used in the calculation, resulting in BMI = 26.8. The analyses were made using the modified Widmark R, and the Widmark equation (see Appendix). In 1986, the beer served at restaurants in Sweden could range between 2.26 and 4.5% (recalculated as volume%) (Johansson, 2008), and the size of “one beer” ranged typically between 33 cl (bottled beer) and 56.8 cl (draft beer, imperial pint). Based on the witness statements and considering these choices of beer served at the time, his most likely range of consumed alcohol amount was between (a) 74 g of alcohol (3^*^33 cl 2.26% beer, 3^*^4 cl 40% liquor and an additional 33 cl 2.26% beer and an additional 4 cl 40% whisky) to (b) 131 grams of alcohol (3^*^56.8 cl 4.5% beer, 3^*^4 cl 40% liquor and an additional 56.8 cl 4.5% beer and an additional 4 cl 40% whisky). For the purpose of exemplifying BAC calculation, the start of alcohol consumption was set to 5.00 PM and the time frame for all ingested alcohol was probably within 1,5 h. By using this practical approach with a simplified time frame, the peak BAC was possibly over-estimated, but the decline was also somewhat over-estimated since the later time-point for the last drink was not taken into consideration. For estimating BAC decline, a time frame of 6 h was assumed, from 5.00 PM to 11 PM. In social drinkers under normal circumstances, BAC decline is constant at ~0.0156 BAC/h (but for heavy drinkers and alcohol-dependent individuals the decline is faster, usually and conservatively estimated at 0.02, but possibly up to 0.035/h) (Winek and Murphy, 1983; Jones, 2010). The volume of distribution for a man of the height and length estimated was calculated with a Modified Widmark R of 0.73 (Seidl et al., 2000) (see Appendix and reference card for calculations). This nuanced BAC calculation showed that the highest vs. lowest estimate of BAC at the witnessing of the crime differed substantially. At the lowest estimate (74 g alcohol and the decline of a heavy drinker) the witness' BAC was 0.00 and at the highest estimate (131 g alcohol and the decline of a social drinker) the BAC was 0.13. Hence, depending on the interpretation of the witness' reported consumption (i.e., amount and time frame), his alcohol experience, and physical appearance, his intoxication status ranged from sober up to being over BAC = 0.13. This BAC-level is within the range where previous studies have found a decreased completeness, but maintained accuracy and reliability, for intoxicated witnesses. In addition, factors such as food intake and individual response to alcohol regarding impairment, stimulation, and sedation must be considered in the discussion of the impact of the estimated BAC on witness performance.

## Discussion

### Practical aspects of interviewing and evaluating reports by intoxicated witnesses

The alcohol-intoxicated witness in the Palme-case was an impartial and passer-by witness to a crime of lethal violence late on a Friday night on his way home from the pub. Since a considerable number of interpersonal violence crimes outside the home occur during similar circumstances (Dingwall, 2006; BRÅ, 2009)—people standing in line in pubs or walking the streets on a night out—it is important for police to know what is especially important to consider regarding interviewing them and evaluating their reports. In this case example, important factors for legal practitioners and for future research on intoxicated witnesses to focus on were highlighted, as well as factors to include in the formula used to estimate BAC to increase the ecological validity of studies within this research field. Using the Palme case as an example, these two areas of application are focused on here, discussing the results for researchers on how to make more nuanced BAC calculations to increase ecological validity and how legal practitioners can apply the present state of research in the field.

### Legal praxis: Intoxication and interview circumstances

The interview transcripts indicated that this intoxicated witness in the Palme-case gave a relatively detailed account of his alcohol consumption (particularly how much alcohol he had consumed, and during what time frame). His details regarding alcohol consumption were reported with more certainty from the beginning of the evening (~4.30–6.00 PM), while he was more unsure of what he drank later on in the evening (i.e., whether or not he drank one whisky). He repeated this uncertainty in Interview 3, then also added the possibility of sipping one more beer after “happy hour”. This follows the familiar pattern of both primary effects and of remembering details more hazily and feeling less certain of one's recollections, during the time period when BAC-level rises (e.g., Pihl et al., 2003; Söderlund et al., 2005), as well as the dose-response relationship to memory (i.e., the higher BAC, the less complete recall). This could also be mirrored in his general expression of uncertainty about how much time had elapsed between different events during the evening. However, in the one instance where his perception of elapsed time is verifiable with objective data (from the ATM surveillance camera), showed that he has made a relatively accurate approximation. Based on his expression of uncertainty regarding what happened during a shorter space of time during which his party left the restaurant, this is also in line with experiencing gray-out/blackout—here in the form of shorter spaces of time during an evening that either is excluded (for some reason) or cannot be explicitly recalled.

This experience is in line with what happens in social drinkers at the low point of his approximated BAC-range, elapsed time before witnessing the crime for a man that was, to the best of our knowledge, a social drinker with no reported drinking problems, but with a probable heavy drinking day at the night in question. However, when he left the ATM, his report seems again uninterrupted by greyout/blackout. The walk his party made from the restaurant to the ATM should have taken ~20 min (according to today's approximation of walking pace by Google maps) during which he did not have time to drink more alcohol. At this time, his BAC was declining, in other words, he was in the phase of the BAC curve where sedation was setting in. In fact, he reported feeling tired and wanting to go home when standing outside the ATM. Considering the sedating alcohol effects in combination with the late hour after a day's work and his reported tiredness, he should have been sub-optimally attentive (due to more reasons than alcohol) when he shortly thereafter walked behind the Palme-couple and the murderer. Indeed, his report on this section of events, before the shots, indicates inattention, as he presumes he is walking behind two women and a man. He also made the interpretation at the time (relying on social stereotypes) of their walking approximately beside each other that they (a) must be a party and (b) that they all three, therefore, were involved in a conversation. It is important to note that he expressively said that he was not sure whether they talked to each other, but since they seemed (to him) to be walking together as a party, he had assumed this. This indicates what has been noted in previous research on similar intoxication levels, an ability to express uncertainty regarding what he/she is less certain of, indicating that a certain degree of metacognition still remains even though alcohol may have lowered this quite a bit.

After hearing the shots, his report (or at least the transcript of it) became more detailed again, including more details regarding for example perceived smells and sounds, but also displaying his unpreparedness for violence, based on his reporting of first thinking the bangs he heard were “Easter crackers” (similar to English “Christmas crackers”). His report then also shows indications of shock (e.g., that he believes that Palme's wife talks in a foreign language after having witnessed Palme fall and he had heard her scream). That he also reported having maintained a flash image of her blood-spattered face when she looked up, could be an effect of shock of this unexpected morbid image either by itself or in connection with his vulnerability due to his intoxicated state. Based on this, it is possible that he experienced more comprehensive problems with information processing/memory encoding in these moments. It is also possible that his problems in reporting this segment were due to a recall issue, for example feeling fear when recalling this and not wanting to think about details. That he experienced fear at that point in that sequence of events is also supported by his reported associations that he reported to have suddenly recalled hearing recent news stories about gang criminality and shootings in central Stockholm and he, therefore, hid in an adjacent doorway. In sum, despite feeling tired, somewhat intoxicated and inattentive and at one point fearful, the witness overall displayed a maintained ability to use several higher-order cognitive functions, could nuance his reported information by adding a degree of certainty and could hold fast to “I don't know” answers over time and repeated questioning.

#### When to interview: The effect of delay and repetition

The witness in the Palme-case was interviewed later on the night of the crime, and allegedly also had reported “all he knew” in a statement almost directly after having witnessed the murder. By being interviewed in this manner, the conditions to report as much as he could remember and to maintain more of this information (here: reporting it consistently and with a certain degree of detail) over the course of interviews, should—according to conclusions in recent research on intoxicated witnesses, have been better than if he had not been interviewed so soon. Of course, this is a conclusion based on his pattern of reported information in the available interview transcripts, and whether this was indeed the case for him cannot be definitely verified. However, based on research on intoxicated witnesses, he should at least have had a better possibility to do this than if the police had waited 1 week to interview him for the first time.

From the interview transcripts, it can be concluded that the witness reported an overall consistent scenario over the course of repeated interviews, even after 13 months. His reports vary in degree of detail over the course of the evening, indicating a pattern that corresponds to his presumed BAC-level. In the interview transcripts, it is also noted that even after repeated questioning, not only within the same interview but also repeated over the course of several interviews, he maintained his “I don't know”-answer regarding details that the police consider important. Due to this being a high-stakes case, it is indeed remarkable that he under such pressured circumstances does not “give in” and engage in speculations, despite acute intoxication and fatigue during the first interview. Therefore, his degree of suggestibility, acquiescence, and compliance seems to have been relatively low, despite intoxication at the time of the witnessed event (Gudjonsson, 2018). Considering the susceptibility of “don't know” answers to leading questions and interrogative pressure, particularly after observing a murder, suggests that this key witness had excellent source monitoring skills, judgment, and resilience (Gudjonsson and Young, 2021; Gudjonsson et al., 2022). In addition, he reported relatively few details regarding the appearance of the perpetrator and, also in this regard, repeatedly declines to give an answer due to “not knowing the answer”. This indicates a selection in line with quantity-accuracy trade-off, that he is sparse with details, only reporting the ones he feels certain of—and also maintains these details over repeated interviews. His direct explanation of not being able to provide answers, even after repeated questioning, demonstrates the resilience of his resistant behavioral responses (Gudjonsson and Young, 2021; Gudjonsson et al., 2022). This provides some evidence that he was a potentially credible witness in the Palme case.

### Researchers: What expert alcohol research can contribute with

The purpose of the BAC-analysis was to not only be able to relate previous research on intoxicated witnesses to this case but also to illustrate a more nuanced BAC-calculation as an option to use in future research on intoxicated witnesses and to accommodate for circumstances interacting with intoxication—thereby contributing to closing the gap between research and praxis. The most important practical aspects related to estimating a BAC-level that emerged from studying the circumstances of the intoxicated witness in the Palme-case were uncertainties regarding factors that probably had influenced BAC-level, such as (1) person-specific factors (e.g., height, weight); (2) exact beverage strength and amount; (3) factors influencing decline of BAC such as time period of ingestion, food ingestion, alcohol experience (i.e., high vs. low consumer); (4) context of observation (incl. maintained and/or divided attention necessary, BAC-curve rising or falling). As a general rule of BAC rise and decline, given ordinary conditions and constant alcohol ingestion, the maximum BAC-level is reached at ~1 h. After 4 h, 50% of maximum BAC-level remains, and after 8 h only 10% remains (Paton, 2005). This is an important rule of thumb for future studies to consider since when considering the design of previous studies, the time aspect has varied. It is also important to note gender differences regarding the pace of rising BAC, where an estimate of 5 units of alcohol ingested during 2 h for men and 4 units in 2 h for women is considered to produce a BAC of 0.08 (niaaa.nih.gov). Therefore, it is important that more naturalistic studies on intoxicated witnesses are conducted and attempt to evaluate the effects of such conditions in synergy with the effects of intoxication. For example, intoxicated witnesses often are subjected to various complicating conditions (e.g., darkness, experiencing other stimuli in parallel such as loud music and/or many people around etc.). As previously mentioned, a considerable number of violent crimes in relation to alcohol consumption outside the home (e.g., at pubs) occur around or after midnight, when it is generally more likely that witnesses experience the sedating effects of alcohol, which will be present in the falling BAC curve rather than the stimulating effects that is present in the rising BAC (Hendler et al., 2013). Hence, in real life, these witnesses are not only affected by intoxication but the sedating effects of alcohol, making their cognitive states more difficult to compare directly to results from the laboratory research conducted so far. To our knowledge, the majority of results on intoxicated witnesses from laboratory studies concern BAC-levels on the curve's rising slope where activating alcohol effects may still be discernible. More inattention, sluggishness, etc. could therefore affect intoxicated witnesses in real life, which should be considered as an important area of future laboratory research (e.g., waiting longer before the first interview, perhaps even several hours, but not allowing the witness to sleep before first interview). Nevertheless, as indicated by the Palme-witness, even at the declining slope of the BAC-curve, witnesses may become more alert again if exposed to stressful circumstances, and thereafter able to increase the level of detail in their report.

**Figure 3:**
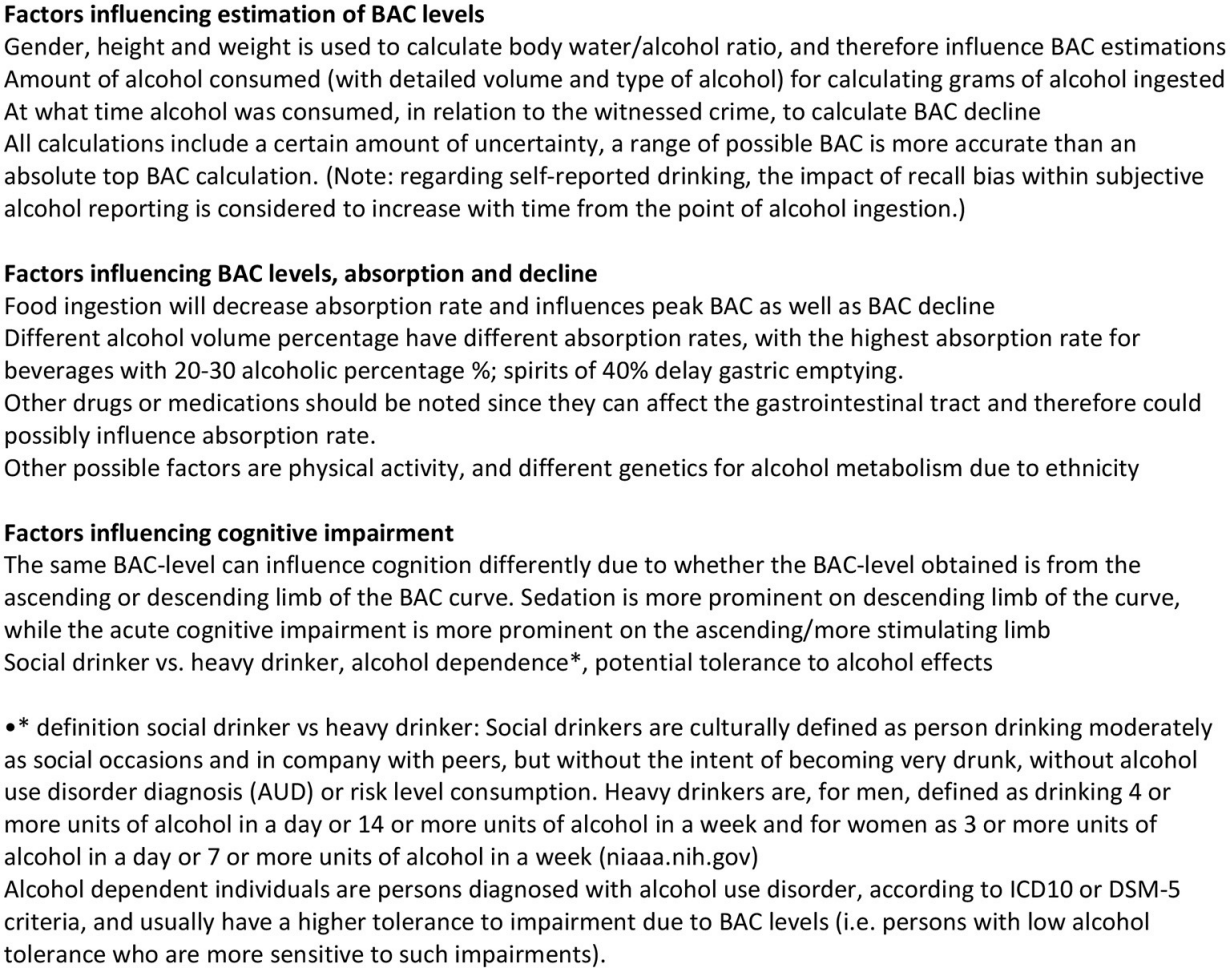
Summary of key points.

The level of impairment at a certain BAC is also highly related to the witness's prior alcohol experience, where experienced/heavy drinkers or alcohol dependent individuals are less impaired at higher BAC-levels (Nestler, 2004). Also, the rate of BAC decline differs in social drinkers vs. heavy drinkers (Winek and Murphy, 1983; Jones, 2010), with heavy drinkers having a faster decline than social drinkers (see Figure 3 for a summary of key points and definition of social drinker /heavy drinker). In previous laboratory research on intoxicated witnesses, this factor has often been checked at the screening stage when selecting participants over/below a certain cut-off, but this has rarely been included as a control variable in analyses. This could result in that what present research considers to result in a high BAC-range is only applicable to a subgroup of participants with relatively low alcohol tolerance (e.g., relatively inexperienced alcohol consumers), while the more tolerant/experienced participants' BACs were substantially lower in both at stimuli exposure and interview.

In summary, several variables affect BAC calculations, the most important individual factors being weight, height, gender as these are key points in the calculations. Several other factors can affect BAC levels, e.g., food ingestion which slower the absorption rate of alcohol. Also, beverages with different alcohol percentages are absorbed differently, whereas beverages with an alcohol percentage of 20–30% create a somewhat faster rise in BAC compared to low percentage beverages (e.g., beer) and high percentage beverages (e.g., strong liquor) (Paton, 2005). The impact of these parameters was however harder to evaluate on both general and individual levels due to idiosyncratic variation and synergies. Nevertheless, the most important information remains (a) the amount and (b) the percentage of the alcohol ingested, as well as (c) at what time the alcohol was consumed, and (d) the drinking experience of the witness. This renders the choice of alcohol type in laboratory research on intoxicated witnesses important, as well as the (previously noted) alcohol tolerance of participants. It also questions current research praxis using only mixed drinks and not also studying the effects of wine, beer, or strong spirits which are very common types of alcohol served in bars and which could potentially produce other kinds of general BAC patterns. Due to such factors, a detailed alcohol interview is important to gather as much knowledge regarding the circumstances as possible to draw more accurate conclusions regarding cognitive impairment in future studies.

## Limitations and future directions

The purpose of this study exemplifies how recent research developments on intoxicated witnesses could be applied to an authentic case where an intoxicated witness reported his observations in a high-profile murder case. This was an illustrating case example and therefore, generalizations to other cases of the exact cited BAC-levels, and decline rate obtained by the present witness, are not possible, although the types of factors considered in the present BAC calculations should be. However, there are some limitations. First, the documented interviews were written as a summary of what the witness had reported (i.e., not documented verbatim) and lacked the exact formulation of questions posed by the police. This made it difficult to evaluate nuances in the reported material, such as how certain the witness appeared in his answers to questions during the interview, something that is very important (both intuitively and explicitly for practitioners) when deciding how trustworthy the reported information is. Nevertheless, the witness at times explicitly stated varying degrees of certainty, as well as was consistent in some key facts which he recanted throughout several documented interviews. This is noteworthy since maintained information over time is considered to be the most trustworthy. These parts of his reports are considerably more detailed could be due to suddenly focused attention due to contextual change, but also to factors in the interview situation, such as the witness' own focus in responding to the questions (e.g., which areas the witness himself/or thinks the police consider to be most important). Regardless of the cause, this is likely the case in all interviews with witnesses, alcohol intoxicated or not. Regarding the BAC levels cited in this study, it is important to note that retroactively calculating BAC-level in the blood is associated with an ~20% margin error (Searle, 2015). Using subjective estimates given by the witness, for example, using time-line follow-back, has been shown to be prone to recall bias and/or uncertainty (Gmel and Daeppen, 2007). That earlier research on intoxicated witnesses has focused on certain levels (often not much higher than BAC = 0.08) can be a point of reference at a group level, but due to varying alcohol absorption and elimination rate among individuals, both researchers and legal practitioners need to consider contextual and individual differences. Regarding future directions, a few things can be noted based on this case study. Although field studies are important, laboratory research on intoxicated witnesses is still needed to simulate alcohol's effects on witness statements in more ecologically valid contexts than has been done previously. Important examples of such contexts are to study the declining BAC-curve (e.g., studying fatigue due to late night observation and BAC from descending limb of curve) or allow for a few hours delay but no sleep before the first interview, or using darkness as a variable of parallel noise/music/distractions when the intoxicated witnesses are exposed to the crime stimulus. This is important knowledge, of how these factors may interact with BAC in creating problems maintaining attention and with memory encoding, to be able to close the gap between research and praxis concerning the handling of intoxicated witnesses.

### Conclusions regarding intoxicated witnesses: What is important to be aware of?

As in the present case example, alcohol intoxicated witnesses to violence are frequently passers-by witnesses. To be in this witness' situation could happen to anyone regardless if he/she has been drinking alcohol or not. To feel unsafe (e.g., due to the unfamiliar and very serious situation they suddenly find themselves in) in combination with (a) alcohol's impairing effects on meta-cognition making them feel less sure of their observations, and (b) social stereotypes that alcohol generally should impair memory, increase the risk for giving a poor report with many “don't know”-answers out of caution, or even worse, guesses in an attempt to accommodate the police or thinking that the police “knows better/more since I was intoxicated”. To be treated with respect and understanding regarding their vulnerable situation (i.e., to have witnessed something frightening while being unsure of how much of their memories they can trust due to knowing they have consumed alcohol) is, therefore, a necessary basis for a comprehensive and reliable report.

Even though the research field on intoxicated witnesses is young, this document analysis of the Palme case—exemplifying how to apply recent research to a real case—was an attempt to bridge the gap between research and practice in the field as well as to inform future research. The analysis highlighted some aspects for legal practitioners to be aware of when interviewing intoxicated witnesses and interpreting the relevance of reported information. The case also highlights the need for interviewers to record verbatim all conversations of witnesses taken at the crime scene and electronic recordings of all police interviews. The results also suggest a structured approach to retrospectively estimating BAC from parameters collected in the interview setting. With a detailed interview regarding key points on alcohol ingestion, the time frame of consumption, and relevant personal details, the range of possible BAC levels could be more specific and more accurate conclusions drawn regarding the intoxicated witness' credibility.

## Data availability statement

The original contributions presented in the study are included in the article/Supplementary materials, further inquiries can be directed to the corresponding author.

## Ethics statement

Written informed consent was not obtained from the individual(s) for the publication of any potentially identifiable images or data included in this article.

## Author contributions

Study design, manuscript preparation, and manuscript editing: MH, AB, HA, and GG. Data analysis: MH (documented interviews, text analysis), AB (alcohol calculations/BAC analyses). Data interpretation: MH and AB. All authors contributed to the article. HA, unfortunately, passed away before the final publication of this article. The remaining authors approved the final submitted version.

## Conflict of interest

The authors declare that the research was conducted in the absence of any commercial or financial relationships that could be construed as a potential conflict of interest.

## Publisher's note

All claims expressed in this article are solely those of the authors and do not necessarily represent those of their affiliated organizations, or those of the publisher, the editors and the reviewers. Any product that may be evaluated in this article, or claim that may be made by its manufacturer, is not guaranteed or endorsed by the publisher.
