# Supplementary material for: What does current science tell us about the accuracy, reliability, and completeness of intoxicated witnesses? A case example of the murder of a prime minister
Source: Front Psychol. 2022 Oct 28;13:982992. doi: 10.3389/fpsyg.2022.982992 (PMC9650999; doi:10.3389/fpsyg.2022.982992)
Supplement: Supplementary file 1 [file Data_Sheet_1.PDF]

*Appendix. Information units reported in respective interviews: Stability of key information points over the course of all conducted interviews.*

**Interview number (date, time span): time relation to the witnessed crime**

**Initial statement at the scene (1986-02-28): No details given.**

**Interview 1 (01-03-1986, 02.25-02.50): Later the same night after having witnessed the shooting**

*Contextual circumstances: the restaurant:*

1. Had been at *Klara* restaurant with his work
2. When he and his company walked away, they walked to a bank (he described the roads taken)
3. Waiting at the bank, he changed his mind and started to go home instead, taking the same road as they had come

*Contextual circumstances: the walk after the Palme-couple:*

4. Saw three persons walking in front of him
5. All approx. same height
6. He thought that they are all together/a company; he thought that they looked like they were having fun and making small talk
7. Walked behind them during at least five meters
8. The person who walks closest to the houses held his arm around what he thought was a woman (walking in the middle)
9. Another woman walked closest to the street
10. Heard two bangs ("like Easter crackers") when the company walked approx. three meters in front of him and he saw the middle person drop down
11. As this happens, he hears the woman closest to the street "screams something in what he perceives is a foreign language"
12. She seemed very surprised when the shots were fired
13. Surmised that the shooter had waited until reaching the crossing since the shots were fired exactly when the company was in the middle of the crossroad.
14. The shooter walked away in a calm pace into a crossing street and disappeared out of view.
15. When he saw this, he quickly hid in a doorway close by.
16. "Seconds after the shooting" a van, probably grey, with three men of which one of them talked on a car phone and they asked him that "he must have seen what happened". "Soon thereafter" the first police cars arrived, he told a female police officer what had happened and after this he went straight home.

*The shooter:*

17. Wore a dark or dark blue, knitted, cap that was rolled up by its edges a couple of times
18. Wore a "dark 'coat-like' garment, that went down to the knee".

**Interview 2 (03-03-1986), 19.30-20.30): Two days after having witnessed the shooting**

First in this documented interview, it is noted that the witness is relatively consistent with his earlier reported information.

*Contextual circumstances: the restaurant:*

19. 15-17 persons from his workplace went to *Klara* restaurant around 5pm where they ate dinner and drank alcohol.
20. Consumed three shots and two-three beers and possibly also a whisky with his coffee; reported subjective feelings of intoxication.

*Contextual circumstances: the walk to the bank:*

21. Reports that "the next time he really remembers is standing outside the bank, looking at his watch and noting that it was 11pm or later"
22. He walked towards another restaurant (*Kåren*) with six persons from the original party, but ending up only walking with three of them since the other three were more intoxicated and "fell behind",
23. At two occasions, the group he walked with stopped and waited for the other three.
24. The second time, the group did not wait until the others actually caught up with them and instead started walking towards the other restaurant but stopped by a bank's ATM.
25. Getting "fed up with the whole thing", he started to walk home; recalling thinking that he could look for the group of co-workers who fell behind.

*Contextual circumstances: the walk after the Palme-couple:*

26. His judgement concerning his degree of intoxication was that “he had clear memory-images of certain things. One of these was that he, in front of him, saw a company of three persons”.
27. He perceived one man (i.e. shooter) and Palme to be of the same approx. height and the woman as somewhat shorter
28. He did not recall them actually talking to each other, but stated that since they walked so close to each other he thought it natural that they were talking to each other.
29. In answer to specific questions of his degree of attention on the three persons, he said that he “naturally” did not look at them the entire time but looked forward to see if he could see his friends from work.
30. In answer to follow up questions, “he couldn’t say how long he was walking behind the company, but that the distance between him and the company was between 5-7 meters.
31. The man who later walked away held his arm around Palme; it was this that had made the witness initially assume that Palme was a woman.
32. In the middle of a crossing, he heard two bangs “like Easter-crackers”, not particularly loud. He looked around to localize the source, until he saw the person in the middle drop down. At the same time, he noticed that the man who had held his arm around the middle person walk away.
33. He did not see a gun. Saw one or two puffs of smoke located close to where the man lay on the ground.
34. When Palme was lying on the pavement and the woman was crouching over him, he “heard how the woman said something in what he perceived was a foreign language. He did not know what was said, but remembered that “when the woman looked up, her face was bloody.”
35. The woman knelt next to the person on the ground; remember feeling scared due to remembering recent events that he had read about in the papers (i.e. violence in the streets due to criminal gangs) and quickly moved into a gateway close by.
36. He noticed later a yellow taxi stopping by the sidewalk and two women or girls run to the man on the ground and later on saw how the police arrives at the scene. He left his name and got to leave.

*The shooter:*

37. “He remembered very well that the person had a roughly knitted cap on, covering the ears and that the cap’s edge was turned upwards into a wide fold”. He is said to be certain of this fact regarding the cap and that he told his wife about this immediately when returning home after witnessing the crime (which the wife confirmed).
38. Furthermore, he said to have “a memory of a long coat and that this coat ended a bit below the knee and was that was flapping. The coat was dark. He could not place the coat on the person who went into Tunnelgatan [i.e. the shooter], it could have been Palme [who was wearing the coat]”.
39. The shooter took two “agile” steps before disappearing from view into the crossing street. He did not perceive the shooter as “disappearing quickly from the scene” but instead “more that he turned and went another way although in an agile manner”.
40. “Did not see a weapon at any point”. “Spontaneously concluded that the man had shot Palme with his left hand while holding his right around Palme’s shoulders”. He could not say anything regarding the shooters build apart from comparative height.

*Other information of relevance:*

41. Recounting the scenario to his wife when returning home, he had mentioned: a) that “it was so strange because a company of three persons walking together and then one of them shoots another and this person drop down and the other just calmly walks away”.
42. His wife had perceived that “he was incoherent in his recounting in a manner [due to] partly had had a chock and partly alcohol intoxicated. She confirmed that the factual information he gave during this interview was “what he told [her] when he came home”.
43. He said that he “during this day” he had talked to all three of the co-workers who walked with him before he witnessed the shooting, but that none of them could remember having met Palme”.

**Interview 3 (25-03-1987), 10.00- ? (no information given); approx. 13 months after having witnessed the shooting**

*Contextual circumstances: the restaurant:*

44. Been to an after work with some colleagues at *Klara* restaurant, where they ate and drank.
45. Described drinking from 16.30 and approx. during one hour thereafter.
46. Consumed three shots (á 4 cl liquor) and three beers, and perhaps also “one small whisky” with his coffee. An addition to the interview made later (dated: 1987-08-07, 13.00pm) the witness reported during a telephone call concerns that it was happy hour between 16.30.18.00 and that he had consumed the alcohol within this time frame. He also added that he may thereafter potentially had been sipping on one beer, but no more.

*Contextual circumstances: the walk to the bank:*

47. He and six others from his company left the restaurant in question around 23.00 to go to restaurant *Kåren*
48. He walked with three others, forming a separate group, since the three others were more intoxicated and fell behind. He described walking the same roads taken as in interview 1.
49. He felt affected by the alcohol he had been drinking during the evening.
50. His company stopped at a bank, where two of them withdrew cash from an ATM boxed in by glass doors [time of withdrawal confirmed by bank records as 23.15 and 23.16].
51. Waiting alone outside, he wondered where the rest were and after a while, he walked away from the doors a bit [not more than 10 meters] to look up the road and possibly see them, but did not although standing there “a good long while”.
52. When his company exited the ATM glass compartment, they “started walking up the road and did not seem to care about him”, and he does not care to stop them and wondered whether he should go home instead.
53. At this point, he may have started to walk but he was not sure of this, when he noticed three persons on the crossing Sveavägen/Tunnelgatan.
54. The person “on the far left lifts his right arm into the air, as when putting their arm around someone.”
55. Directly thereafter, “he hears two bangs in succession, although not particularly loud”.
56. He thought when he saw the arm movement that the man on the left formed a company with the two others (here: women).
57. The three were of approx. the same height, darkly dressed and they “seemed to belong together in some way”.
58. He saw “the middle person fall and how [the shooter] calmly moved to the left and walked into Tunnelgatan eastbound and disappeared from [his] view, but before walking into this street, the man turned 90-180 degree angle.
59. The man who fell stayed “lying down almost in the middle of Tunnelgatan [i.e. shot while crossing this street] and the woman on the far-right bent down towards this person and exclaimed something that he perceived as incomprehensible in a strange language. He could not remember what”.

*The shooter:*

60. “Darkly dressed, in a ¾-long flapping coat-like garment”, and “dark pants” and “unknown types of shoes but he had a notion that the soles had a light color”, and “everything around the neck gave a dark impression”.
61. “Dark blue or black knitted cap with the edge rolled up in such a manner that it stood out and was clearly visible. The cap went quite far down the neck and the collar of the coat also hid hair and the neck”. He also said that he thought the cap was of a vertical “rib knit”.
62. Could not give any information regarding body type, but “had a feeling that the man moved smooth/agile”.
63. Apart from that, he could not say anything regarding “glasses, beard, moustache, skin color etc.”.
64. He never saw the gun or if the man was carrying anything else.

*Other information of relevance:*

65. At the start of the interview, the witness was asked to, in detail, recount for his actions and observations regarding Friday night the 28$^{th}$ of February, 1986
66. The observation time was short, “his observations of the man only lasted for a couple of seconds”
67. He did not remember having seen either of these three persons before the two bangs.
