# Supplementary material for: What does current science tell us about the accuracy, reliability, and completeness of intoxicated witnesses? A case example of the murder of a prime minister
Source: Front Psychol. 2022 Oct 28;13:982992. doi: 10.3389/fpsyg.2022.982992 (PMC9650999; doi:10.3389/fpsyg.2022.982992)
Supplement: Supplementary file 2 [file Data_Sheet_2.pdf]

BAC REFERENCE CARD

Estimating a witness' Blood Alcohol Concentration (BAC)

Witness personal data

Name | Date of birth

Gender: Male ☐ Female ☐ Weight (kg): ________ Height (cm): ________

Time of interview: (date and time) | Time of witnessed crime: (date and time)

Breath Alcohol Concentration at interview (‰)

Alcohol details

Type of beverage (% alcohol)	Volume (l)	Time of ingestion	Calculated grams of 100% alcohol	**Estimated BAC at witnessed situation***
				**Estimated BAC at interview***
				*formula BAC on back page

formula for calculating grams of 100% alcohol:
volume (L) x percentage ethanol (%) x (factor for density of ethanol) 7.89 = alcohol in grams

Witness is considered to be (drinking experience defined on back page):

- Inexperienced drinker (alcohol elimination rate 0.012 BAC/h) ☐
- Social drinker (alcohol elimination rate 0.0156 BAC/h) ☐
- Heavy drinker (alcohol elimination rate 0.02 BAC/h) ☐
- on rising BAC curve (sedative component more prominent) ☐
- on falling BAC curve (stimulative component more prominent) ☐

BAC = grams of alcohol per 100mL blood, or %
Permille = grams of alcohol per 1L blood, or ‰
e.g. BAC of 0.08 equals permille of 0.8

© de Bejczy & Hildebrand Karlén

BAC REFERENCE CARD

Estimating a witness' Blood Alcohol Concentration (BAC)

Definitions and guidelines

One unit of alcohol is defined as 12-14 grams of 100% alcohol.
Examples of one unit of alcohol are:

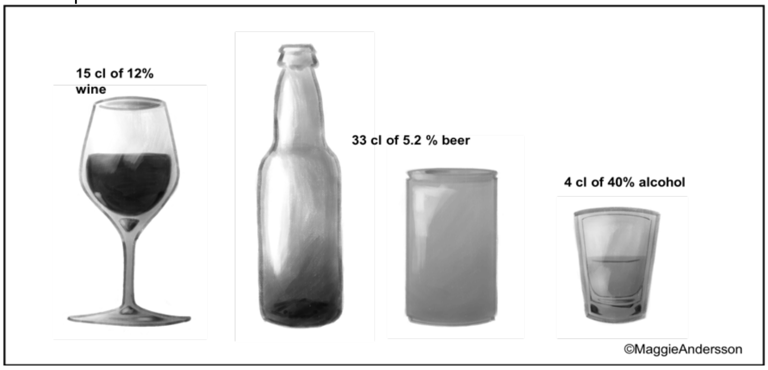

Definition of **social drinking** is:
Drinking moderately at social situations/in company of others, without becoming intoxicated and without a pattern of risk consumption

Definition of **risk consumption/heavy drinking** is:
For men: 4 alcohol units or more in a day or 14 alcohol units or more in a week
For women: 3 alcohol units or more in a day or 7 alcohol units or more in a week

To calculate BAC the **Widmark formula** is used;

(Alcohol consumed in grams/(body weight in grams)*R)*100
R= Widmark constant is 0.55 for females 0.68 for males

For more accurate estimation of distribution of alcohol in body water volume, the **modified R (rMI)** can be used instead of the R in the formula above

Formula to calculate rMI: 0.31608-0.004821*body weight(kg)+0.004632*body height(cm)

For calculating **elimination of BAC** the following formula is used, where the elimination rate is estimated based on drinking habits (for elimination rates, see front page)

t(time elapsed since start of drinking in hours)*elimination rate

Example

Witness started drinking at 6 pm and consumed 2 beers (33 cl, 5.2 %) and 2 glasses of wine (15 cl, 12 %) during the evening. At 11pm he witnessed a crime and at 12pm he was interviewed. Time between alcohol consumption start (6pm) and crime (11pm)= 5h. Male, 80 kg, 175 cm, social drinker (elimination rate 0.0156).
Alcohol consumed in grams: (0.33*5.2*7.89)*2+(0.15*12*7.89)*2= 27+28= **55g**
BAC at maximum peak: (55/(80000*0.68)*100)= **0.1**
BAC elimination over 5 hours: 5*0.0156= **0.078,** BAC elimination over 6 hours: 6*0.0156= **0.0936**
BAC at time of crime: 0.1-0.078= **0.022,** BAC at the time of interview: 0.1-0.0936= **0.006**

Reference modified Widmark R: Seidl, S., Jensen, U., & Alt, A. (2000). The calculation of blood ethanol concentrations in males and females. *Int J Legal Med, 114*(1-2), 71-77. doi:10.1007/s004140000154
reference elimination reate: Jones, A. W. (2010). Evidence-based survey of the elimination rates of ethanol from blood with applications in forensic casework. *Forensic Sci Int, 200*(1-3), 1-20. doi:10.1016/j.forsciint.2010.02.021

© de Bejczy & Hildebrand Karlén
